# Residents’ Self-Reported Health Effects and Annoyance in Relation to Air Pollution Exposure in an Industrial Area in Eastern-Estonia

**DOI:** 10.3390/ijerph15020252

**Published:** 2018-02-02

**Authors:** Hans Orru, Jane Idavain, Mihkel Pindus, Kati Orru, Kaisa Kesanurm, Aavo Lang, Jelena Tomasova

**Affiliations:** 1 Institute of Family Medicine and Public Health, Faculty of Medicine, University of Tartu, Ravila 19, 50411 Tartu, Estonia; janemari@gmail.com (J.I.); Mihkel.Pindus@ut.ee (M.P.); 2 Department of Public Health and Clinical Medicine, Umea University, SE-901 87 Umea, Sweden; 3 National Institute for Health Development, Hiiu 14, 11619 Tallinn, Estonia; 4 Institute of Social Sciences, University of Tartu, Lossi 36, 51003 Tartu, Estonia; Kati.Orru@ut.ee; 5 Estonian Environmental Research Centre, Marja 4d, 10614 Tallinn, Estonia; Kaisa.Kesanurm@klab.ee; 6 Institute of Biomedicine and Translational Medicine, Faculty of Medicine, University of Tartu, Ravila 19, 50411 Tartu, Estonia; Aavo.Lang@ut.ee; 7 Estonian Health Board, Paldiski 81, 10617 Tallinn, Estonia; Jelena.Tomasova@terviseamet.ee

**Keywords:** oil shale, shale oil, particulate matter, phenol, benzene, respiratory, cardiovascular

## Abstract

Eastern Estonia has large oil shale mines and industrial facilities mainly focused on electricity generation from oil shale and shale oil extraction, which produce high air pollution emissions. The “Study of the health impact of the oil shale sector—SOHOS” was aimed at identifying the impacts on residents’ health and annoyance due to the industrial processing. First, a population-wide survey about health effects and annoyance was carried out. Second, the total and oil shale sectors’ emitted concentrations of benzene, phenol, and PM_2.5_ were modelled. Third, the differences between groups were tested and relationships between health effects and environmental pollution studied using multiple regression analysis. Compared to the control groups from non-industrial areas in Tartu or Lääne-Viru, residents of Ida-Viru more frequently (*p* < 0.05) reported wheezing, chest tightness, shortness of breath, asthma attacks, a long-term cough, hypertension, heart diseases, myocardial infarction, stroke, and diabetes. All health effects except asthma were reported more frequently among non-Estonians. People living in regions with higher levels of PM_2.5_, had significantly higher odds (*p* < 0.05) of experiencing chest tightness (OR = 1.13, 95% CI 1.02–1.26), shortness of breath (1.16, 1.03–1.31) or an asthma attack (1.22, 1.04–1.42) during the previous year. People living in regions with higher levels of benzene had higher odds of experiencing myocardial infarction (1.98, 1.11–3.53) and with higher levels of phenol chest tightness (1.44, 1.03–2.00), long-term cough (1.48, 1.06–2.07) and myocardial infarction (2.17, 1.23–3.83). The prevalence of adverse health effects was also higher among those who had been working in the oil shale sector. Next to direct health effects, up to a quarter of the residents of Ida-Viru County were highly annoyed about air pollution. Perceived health risk from air pollution increased the odds of being annoyed. Annoyed people in Ida-Viru had significantly higher odds of experiencing respiratory symptoms during the last 12 months, e.g., wheezing (2.30, 1.31–4.04), chest tightness (2.88, 1.91–4.33 or attack of coughing (1.99, 1.34–2.95).

## 1. Introduction

Eastern Estonia or Ida-Viru is a north-eastern county in Estonia bordered by the Finnish Gulf to the north, the Narva River and Russia to the east, and Peipsi Lake to the south. The county has a long tradition of industries focused on oil shale (mining, energy generation, and chemical production). The extraction of oil shale began in Estonia in 1916. The annual output peaked in 1980 with 29.7 million tons, after which it started to decrease due to the construction of a nuclear power station in Sosnovyj Bor and decreasing electricity exportation to Russia [1]. By 2003, oil shale extraction had fallen to one-third of the peak level, but from that time onwards the output has been increasing. In 2017, the projected output will be around 20 million tons, the maximum amount allowed to be mined by the Estonian government [2]. 

The oil shale industry employed 6400 people in 2016 and is one of the Estonia’s largest employers [3]. However, oil shale mining and electricity generation processing is emitting excessive amounts of sulphur dioxide (SO_2_), particulate matter (PM_10_), and nitrogen oxides (NO_X_), as well as various other industrial pollutants, such as benzene and phenols released in oil extraction, and trace elements [4]. Furthermore, the workers are exposed to hazards due to the physical and chemical characteristics of oil shale and its derivatives [5]. Health impacts related to the oil shale sector were extensively analysed in Estonia up to the early 1990s, when the environment’s condition and health indicators of the inhabitants, and their interrelations, were thoroughly studied [6]. Since then, only occupational health risks such as exposure to exhaust gases during mining, and polycyclic aromatic hydrocarbons and benzene in the shale oil industry have been studied [7,8,9,10,11,12], and there have been no studies on the health effects of the oil shale industry during the last 15–20 years.

In industrially contaminated areas, many different human health effects have been documented, such as increased mortality and morbidity rates, hospital admissions, and reproductive outcomes [13,14,15]. In Italy, an analysis of 44 industrially contaminated sites showed an excess of deaths [16] and an analysis of 17 sites showed an increase in the incidence of cancer [17]. In the area surrounding a large oil refinery, thermoelectric power plant, and petrochemical plants in Gela, Italy, people are at a high risk of lung cancer, and non-malignant respiratory and genitourinary diseases [18]. In Taranto, close to a large steel plant, a refinery, the harbour, and waste dumps, an excess risk of total deaths, all kinds of cancers, lung cancer, and cardiovascular and respiratory diseases, both acute and chronic, have been reported [19]. In areas of similar character in the suburbs of Rome, increased cardiovascular and respiratory hospital admissions, and some forms of cancer (pancreatic, breast, laryngeal) have been documented in relation to H_2_S, PM_10_, and SO_X_ exposure [20]. 

Even though the Estonian oil shale industry is relatively unique, refining shale oil is considered to have health effects similar to other refining operations [5] and thus studies of the people living in the vicinity of refineries continue to be of high relevance. However, the studies conducted over the past 20 years show mixed results in terms of the air pollution and health impacts caused by oil chemical plants. Regarding carcinogenicity, in some cases higher incidences of lung cancer, throat cancer, and liver cancer have been found in industrial areas worldwide [21,22,23,24]. Furthermore, the incidence of bladder cancer has been higher in areas of petrochemical industry (possibly due to exposure to benzene) [25] and a higher risk of leukaemia among older persons living in polluted areas for extended periods has been found [21]. In other cases, no relationships have been found between the petrochemical industry and leukaemia, lymphoma, and blood or central nervous system tumours [26,27]. Studies have also shown that people living within 2 km of a plant, as well as people who had been living close to a plant for at least 10 years, had a higher risk ratio in terms of various respiratory diseases [28,29]. However, breathing difficulties, chronic asthma, and asthma-related deaths did not increase among women and children living near an industrial complex [30,31]. An increased risk of pulmonary fibrosis and airway obstruction among oil shale miners has been shown [32], but no other cancer risk increase besides skin cancer have been found [33]. Air pollution in industrial areas may also affect children and foetuses. For example, it was found that living near coal power plants had a negative impact on the development of lung function in children, even though the air pollution of the area did not exceed local standards [34], which suggests they are not strict enough. For both foetal chromosome damage and preterm birth, the risk was higher for women aged 35 years or older who had been living in industrially polluted areas for an extended period [35].

Apart from the direct health effects caused by exposure, the mere perception of pollution may cause annoyance. The concept of annoyance is complex and can be considered a perception, an emotion, an attitude or a mixture of these [36]. Annoyance has also been identified as a useful signal for potential health effects of pollution in a community [37,38]. As for the mechanisms behind annoyance and symptoms, perceived pollution is likely to play an important role. Perception of air pollution is predominantly based on visual and chemosensory cues, and has been shown to mediate between environmental exposure and health [39]. If the recognised source is perceived as unpleasant, it is likely to have a negative impact on health, e.g., evoke annoyance, worry, or disgust as an exacerbation mechanism [40]. Another factor that may lead to annoyance, health symptoms, and possibly to disease, is worry due to health risk perception. Belief that a certain chemical/physical exposure is hazardous (irrespective of whether it is hazardous or not), and the worry and stress this evokes, has been shown to contribute to health symptoms [37,41]. This is likely to be explained by mechanisms such as stress-induced inflammation and expectations. Aspects that may modify annoyance include social status and the level of perceived control a person has over one’s wellbeing. For example, people with a low level of education and members of minority groups can be more annoyed [42,43]. This annoyance has also been explained by an incapacity to cope with risks related to one’s physical or psychological wellbeing [44].

When it comes to specific sources of pollution, industrial facilities and large boiler houses significantly contribute to ambient air pollution concentrations, however traffic remains the largest contributor of pollution in general in Europe [45,46]. In Estonia, industrial emissions play important role only in Ida-Viru County [47]. Locally generated pollution is coupled with air pollution from other regions and countries [48]. The highest pollution concentrations have been recorded in Tallinn and Tartu, and PM_10_ concentrations were also high in Kohtla-Järve and Narva (NO_2_ concentrations were lower in the towns of Ida-Viru County because of lower traffic intensity). The decrease in life expectancy was highest in larger towns, such as Tallinn, Tartu, Narva, Pärnu, and Kohtla-Järve, and somewhat higher in Ida-Viru County in general [49]. Epidemiological studies in Estonia have shown a link between levels of particulate matter from traffic and the occurrence of heart symptoms [50], and a higher risk of developing heart disease among those living near high-traffic streets and roads has been found [51]. A time-series study of particulate levels in Tallinn over 2004–2011 showed that mortality rates increased the next day after an air pollution episode, despite the relatively low levels of pollution in general [52]. 

Based on earlier studies, the effect of different air pollutants on health conditions in cities are clear; however, information on the health effects of air pollution in the industrial areas of Estonia is limited. Therefore, the current study aimed to identify the air quality situation in Ida-Viru County (the region with the largest air polluting industry in Estonia) and people’s annoyance regarding air pollution, in order to study the relationships between industrial pollutants and the health of local residents. 

## 2. Materials and Methods

The epidemiological investigation “Study of the health impact of oil shale sector—SOHOS” was a cross-sectional survey, consisting of a health questionnaire for residents, and air quality monitoring and modelling data. The study was part of the material for National Development Plan for the Use of Oil Shale 2016–2030. The cross-sectional study of the people living in three different counties was approved by the Research Ethics Committee of the University of Tartu (231/T-10).

### 2.1. Questionnaire Study

First, a postal questionnaire was sent in 2014 and 2015 to 2097 residents aged 18–70 years old living in Ida-Viru (Eastern Estonia) County. Second, questionnaires were sent to a reference group of 403 individuals aged 18–70 years living in neighbouring Lääne-Viru (west) County and to 2750 individuals aged 18–40 years old living in Tartu (Southern Estonia, with no industrial air pollution). All participants were randomly selected by the Estonian Population Registry according the age and ethnic structure of the area. Third, the information collected during a similar earlier survey entitled “Respiratory Health in Northern Europe—RHINE III”, carried out in 2011 and 2012 in Tartu [53], was included into the Tartu reference group. RHINE III consisted of 2231 participants from Tartu aged 40–65 years old, of whom 1370 responded to the questionnaire.

All participants received a postal questionnaire about their socio-economic situation, general health, respiratory symptoms, chronic diseases, indoor and outdoor environment, working history, and health behaviour (see details of the questions in Appendix A). The SOHOS questionnaire was largely based on the RHINE III questionnaire, but additional questions were added according to the needs of the current study. According to their self-defined ethnicity in the population registry, the questionnaire was sent either in Estonian or Russian. The respondents sent the questionnaire back in a closed, pre-paid envelope. The questionnaire was posted twice and on a third occasion a reminder was sent. Those who had an e-mail address in the population registry received the reminder by e-mail, those without e-mail got theirs by regular mail. With the reminder, participants also received the possibility to fill in the questionnaire online.

### 2.2. Air Quality Monitoring and Pollution Dispersion Modelling

**Figure 1:**
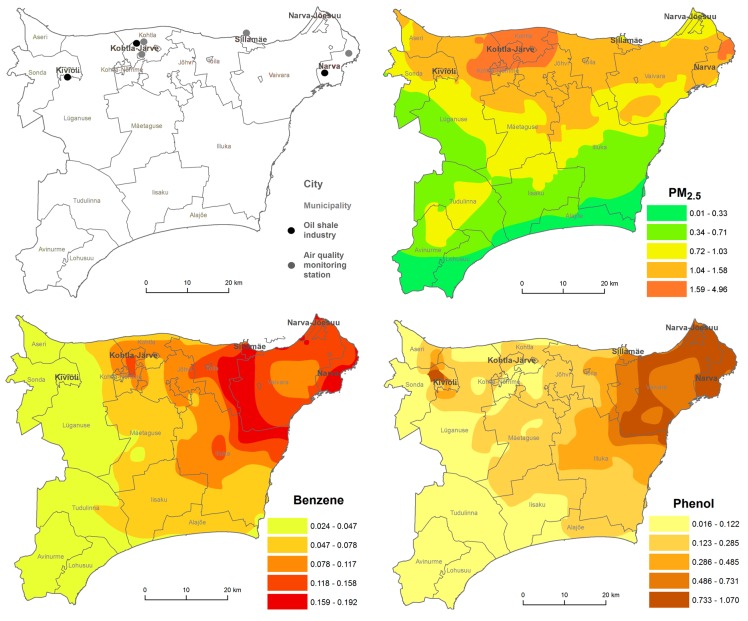
Oil shale industry facilities, air quality monitoring stations and annual average concentrations (μg/m^3^) of fine particles (PM_2.5_), benzene, and phenol in the ambient air of Ida-Viru County.

The study involved an analysis of monitoring data from Ida-Viru County in 2001–2013, followed by the modelling of pollutant levels to quantify the exposure to pollution of the population throughout the region. The monitoring data was retrieved from the Estonian Environmental Research Centre, responsible for air quality monitoring in Estonia. Regular monitoring takes place in Kohtla-Järve, Narva and Sillamäe. The shale oil extraction factories are situated in Kohtla-Järve, Kiviõli, and near Narva. The electricity generation plant is also situated in Narva (Figure 1).

Annual mean concentrations of phenol, benzene, and PM_2.5_ in 2013 were modelled in 1 × 1 km grids in Ida-Viru County using an Eulerian air quality dispersion model that was part of the Airviro Air Quality Management System (SMHI, Sweden; http://airviro.smhi.se), see the detailed description in the Airviro Documentation [54]. Airviro is a widely used web-based air pollution data management tool that uses data on air emissions, measured levels of air pollution, and measured meteorological variables [49]. The industrial pollutant emissions were retrieved from the ambient air emission database OSIS2013, which consists of annual emissions (tons per year) as reported by companies and confirmed by the Estonian Environmental Board. Transport-sector emissions came from the database Traffic2007 and domestic heating emissions from the local heating database. The modelled concentrations were validated with monitored levels from monitoring stations in Ida-Viru County. For the visualization, the model output was generalized and classified into five classes using ArcGIS scripting environment ArcPy. The obtained annual concentrations of modelled pollutants per grid cell were linked with the geo-code of each respondents’ home address in ArcGIS. 

### 2.3. Statistical Analysis

According to their home address, the respondents were divided into three groups: residents of Ida-Viru County, Lääne-Viru County, or Tartu. According to the home address, respondents’ prevalence of respiratory symptoms, chronic diseases, risk perception, and socio-economic indicators were calculated. The differences between the three groups were tested with a chi-square test. Furthermore, the differences between genders, ethnicities, and employed/un-employed in the oil shale sector, were tested.

The relationships between self-reported health effects and modelled pollutant concentrations at the respondents’ home addresses were studied using logistic regression analysis in Stata 12.1 (StataCorp, College Station, TX, USA). The regression models were adjusted for possible confounders such as gender, age, body mass index (BMI), environmental tobacco smoke (ETS), smoking history, and income per family member.

## 3. Results

Altogether 2127 people responded to the SOHOS questionnaire (a response rate of 40.7%). The response rate was highest in Lääne-Viru County (45.4%), followed by Ida-Viru County (40.8%), and Tartu (39.8%). Among the Ida-Viru respondents, 9.8% used the possibility to fill in the questionnaire online. In Lääne-Viru County, this proportion was 6.0% and in Tartu 13.6%. The socio-demographic characteristics of the respondents in SOHOS and RHINE III are given in Table 1. In general in Ida-Viru County Estonians, females and older people tended to answer more frequently (among non-respondents there were 19.7% Estonians, 47.4% females and 19.6% 58–67-year-olds).

### 3.1. Prevalence of Respiratory Symptoms and Chronic Health Effects

The analysis showed that the residents of Ida-Viru County complained significantly more frequently (*p* < 0.05) about wheezing, chest tightness, shortness of breath, asthma attack, long-term cough, hypertension, heart disease, myocardial infarction, stroke, and diabetes compared to residents of Tartu and even compared to residents from neighbouring Lääne-Viru County when it came to wheezing (Table 2). Men living in Ida-Viru County suffered significantly more frequently (*p* < 0.05) from chronic obstructive pulmonary disease, wheezing, and heart attacks than women, and men in reference areas (data not shown). The analysis also identified significant differences between ethnic groups, with non-Estonians suffering more from most of the health complaints and chronic diseases (Table 2).

Besides the living environment, as the working environment also influences the development of diseases, the prevalence of diseases were compared between individuals who had and had not worked in the oil shale sector. It appeared that those who had worked in the oil shale sector (*n* = 241), compared to those not been working (*n* = 540), had significantly more frequently (*p* < 0.05) experienced wheezing (32.8% vs. 30.0%), chest tightness (33.3% vs. 27.4%), hypertension (37.3% vs. 29.8%), heart disease (17.4% vs. 12.6%), cardiac infarction (8.7% vs. 5.9%), stroke (5.0% vs. 2.2%), and diabetes (10.4% vs. 8.3%).

### 3.2. Monitored and Modelled Air Pollution Levels in Ida-Viru County

According to urban ambient air surveillance, the highest number of exceedances of Estonian limit values was related to phenol as a product of the thermal processing of oil shale. The number of exceedances (daily average limit is 3 μg/m^3^) had decreased from 122 in 2004 to 43 in 2013 at the three measuring stations in Ida-Viru County. The annual average phenol concentration in 2013 was 1.12 μg/m^3^ at Järveküla Road, 1.12 μg/m^3^ at Kalevi Street, and 1.3 μg/m^3^ in Narva (Figure 1). The hourly average limits for H_2_S concentration in Kohtla-Järve were exceeded most often in 2007 (230 times); over the following years such occasions remained in the range of 40–50 times, and had decreased to 16 occasions by 2013. The temporal variation of some pollutants can be seen in Appendix B.

Regarding modelled annual average air pollution concentrations in Ida-Viru County, the highest levels of PM_2.5_ appear in the Kohtla-Järve region, the highest levels of benzene in the Narva region, with a slight increase also in Kohtla-Järve region, and the highest levels of phenol in the Narva region, with a slight increase also in the Kiviõli area (Figure 1).

In general the model behaved rather well compared to measured values in monitoring stations (see Appendix B for details). The temporal variation of the model was very good, but it somewhat underestimated the PM_2.5_ and phenol levels in Narva and largely underestimated benzene levels in Sillamäe. Also there appeared to be spatial variation, as the concentrations were higher close to oil shale industry facilities. However, the variations were different for different pollutants and places, e.g., close to Kiviõli there were high concentrations of phenol, but low concentrations of benzene and vice versa in Kohtla-Järve.

### 3.3. The Relationship between Air Quality and the Health Situation in Ida-Viru County

People living in the quartile with the highest levels of fine particles (PM_2.5_) had 1.13 (95% CI 1.02–1.26), 1.16 (95% CI 1.03–1.31) and 1.22 (95% CI 1.04–1.42) higher odds of reporting chest tightness, shortness of breath, and asthma attacks respectively, compared to residents in the quartile with the lowest levels (Table 3). People living in areas with the quartile with the highest levels of benzene had 1.98 (95% CI 1.11–3.53) higher odds of having had a myocardial infarction in the past and people living in the quartile with the highest levels of phenol had increased odds of chest tightness (1.44, 95% CI 1.03–2.00), long-term cough (1.48, 95% CI 1.06–2.07), and myocardial infarction (2.17, 95% CI 1.23–3.83) (Table 3). Sensitivity analysis among the people who have been working in oil shale sector showed increase in ORs, also differences appeared between Estonians and non-Estonians (see details in Appendix C along with crude analysis).

### 3.4. Annoyance by Socio-Demographic Group and Relationships with Health Complaints

The prevalence of high annoyance due to air pollution (respondents who rated 7–10 on a 10-point scale, ranging from no annoyance (1) to very high annoyance (10)) significantly differed between the regions (Appendix D). Respondents (both genders) from Ida-Viru County were significantly more annoyed by air pollution compared to respondents from Lääne-Viru and Tartu Counties. As for differences due to age, there were significant regional differences in annoyance among 28–37- and 38–47-year-olds. In these age groups, Ida-Viru residents were significantly more annoyed by air pollution compared to those of the other regions.

As for the effects of education, compared to the other regions, in Ida-Viru County there were significantly more respondents annoyed by air pollution among people with a secondary or higher education (Appendix D). As for the effect of ethnicity, there were no regional differences in frequency of annoyance among non-Estonians. However, compared to other regions, in Ida-Viru County Estonians were significantly more annoyed than non-Estonians (Appendix D). As per the effect of income, the differences in annoyance appeared only among the highest income group, with wealthy people in Ida-Viru more frequently annoyed, compared to those in Lääne-Viru and Tartu.

Regarding health, people with good health were more frequently annoyed by air pollution in Ida-Viru County, compared to the other regions (Table 4). When it came to health complaints among annoyed people per region, annoyed people had significantly more frequent chest tightness, wheezing, allergic rhinitis, and asthma in Ida-Viru County, compared to those of the other regions.

We clarified factors explaining the annoyance with air pollution in Ida-Viru County and reference regions using logistic regression. In base models predicting annoyance, we included gender, age, education, income, smoking history and self-assessed health status. When considering all regions together, compared to being a representative of Tartu, being a representative of Ida-Viru County significantly increased the odds 1.66 (1.18–2.31) of being annoyed, but being from Lääne-Viru did not increase the odds for annoyance. Table 4 presents the analysis results for specific regions. In the base model, compared to women, men had 0.64 (95% CI 0.42–0.97) lower odds of being annoyed in Ida-Viru County, but also in Tartu. The odds of being annoyed increased by 1.59 (95% CI 1.21–2.08) when the subjective health assessment decreased by one degree on a five-point-scale in Ida-Viru, similar odds appeared in reference regions. Further, we added the possible predictors of annoyance to the base model one-by-one. No effect was seen between the modelled pollutants and annoyance in Ida-Viru County. Due to a lack of data, we could not calculate the effect of pollutants for other regions. The odds of being annoyed decreased by 0.35 (95% CI 0.28‒0.44) if the perceived risk from air pollution to one’s health decreased by one degree on a five-point-scale in Ida-Viru County, and similarly in reference regions. The odds of being annoyed did not increase significantly depending on the ethnic background and the experience of employment in oil shale industry in the Ida-Viru and Lääne-Viru region, but the ethnicity was a significant predictor in Tartu.

In all regions, the odds of being annoyed significantly increased when a person had chest tightness. In Ida-Viru region, the odds of being annoyed significantly increased when a person had symptoms such as wheezing without a cold, attacks of coughing and diagnosed wheezing. In Tartu, the odds of being annoyed increased when a person had shortness of breath, attacks of coughing, wheezing and a long-term cough. The odds of being annoyed did not significantly increase when a person had cardiovascular diseases.

## 4. Discussion

The current study was carried out to identify the air quality situation in Ida-Viru County and people’s annoyance regarding air pollution, as well as to study the relationships between industrial pollutants and the health of local residents. Our cross-sectional study based on a large sample, shows that compared to the control groups from non-industrial areas in Tartu or Lääne-Viru County, Ida-Viru County residents more frequently reported wheezing, chest tightness, shortness of breath, asthma attacks, a long-term cough, hypertension, heart diseases, myocardial infarction, stroke, and diabetes. People living in regions with higher levels of PM_2.5_, benzene, or phenol had higher odds of experiencing chest tightness, shortness of breath, or an asthma attack during the previous year and a long-term cough or myocardial infarction in the past. The prevalence of adverse health effects was also higher among those who had been working in the oil shale sector.

An increased prevalence of almost all cardio-respiratory diseases (except asthma) and diabetes in Ida-Viru County reflects trends from earlier studies made approximately 15 years earlier, where they observed an increase in respiratory symptoms, such as wheezing and crackling in Narva compared to Tallinn and the Western islands [55]; whereas there were no significant differences in asthma incidence among the different regions of Estonia [56]. In our study, we observed that people living in Ida-Viru County in areas with higher levels of PM_2.5_, benzene, or phenol, had significantly higher odds of reporting several respiratory diseases and myocardial infarction, but no other cardiovascular disease or diabetes. Similar relationships were documented in the same region more than 25 years ago by Etlin [6].

The increased risk of respiratory diseases has been shown in industrial areas in China, if residents were living close to a factory or chimney [57], as well as among residents living in high-industry areas in Port Adelaide, Australia [58]. In a Canadian study, Fung et al. showed an increased risk of respiratory as well as cardiovascular disease in industrial cities compared to a reference city, with higher standardized hazard ratios among women [59]. In a recent ecological study of an industrial area in France, Pascal et al. found a higher risk of hospitalization due to myocardial infarction, but no higher risk of hospitalization due to respiratory diseases or cancer [60]. In contrast in England and Wales, Aylin et al. did not show any increased risk of hospitalization due to cardio-respiratory diseases among a population living near coke works [61]. However, a high prevalence of diabetes has been shown in an industrial area of southern Poland [62]. If we compare our finding with existing studies, there is a very significant amount of evidence of the adverse health effects of PM_2.5_ on the cardio-respiratory system [63,64] and as a cause of diabetes [65,66]; there is substantially less evidence of the effects upon health of benzene [67,68] and phenols [69,70], especially when in ambient air, often because of a lack of monitoring data to enable epidemiological studies. 

The study shows that up to a quarter of the residents of Ida-Viru County in eastern Estonia are highly annoyed about air quality. Compared to Tartu and Lääne-Viru regions, the annoyance in Ida-Viru was higher even after controlling for the effects of socio-economic status and subjective health assessment. The mere perception of pollution may cause annoyance and health symptoms as a protective mechanism. However, at the current relatively low concentrations, benzene, phenol and particulate matter may not evoke strong enough odour sensations to activate the chemosomatosensory system, leading to eye irritation and a pungent nasal sensation that could lead to feelings of annoyance in the Ida-Viru region. Worry due to health risk perceptions, i.e., the belief that the exposure is hazardous, may lead to annoyance and health symptoms [37,38,41]. Perceived risk from air pollution to personal health significantly contributes to annoyance from air pollution in all studied regions, regardless of the actual pollution levels. 

Annoyance from environmental exposure has been suggested to serve as an early warning signal of illness [71]. Analysis proved that annoyance from air pollution is related to subjective health assessments as well as to respiratory symptoms, e.g., chest tightness, wheezing or cough. This suggests that annoyance may lead to health complaints. However, the relationship may also work the other way around: symptoms may sensitise individuals to pay more attention to air pollution. Nevertheless, the fact that annoyed Ida-Viru people have more frequent respiratory problems compared to people in other regions, suggests that annoyance is related to the objective conditions in the Ida-Viru region.

As for the other factors mediating the relationship between air pollution and annoyance, women have higher odds to be annoyed by air pollution, as also noted in earlier research [58]. Ethnic background, i.e., being a member of the majority group of Estonians or the minority group of Russians, income, and education do not significantly affect the odds of being annoyed. Thus, in Ida-Viru region, socio-economic vulnerability is not a strong enough differentiating factor in terms of annoyance from air pollution in the Ida-Viru region. Nevertheless, such differences have previously been found in other social contexts [56,59].

The analysis showed a clear problem of industrial pollutants including benzene, phenol, and hydrogen sulphide. The main source of industrial emissions of benzene and phenol are oil shale processing and energy production. Thanks to the toughening of environmental protection requirements and investment in new technologies and treatment facilities, the number of exceedances has decreased. Also, the concentrations of benzene, polycyclic aromatic hydrocarbons, and heavy metals in the ambient air have considerably decreased compared to previous studies [72]. However, the exceedances that still occur need proper addressing by the industry and regional authorities. Sometimes the problems have not been even fully recognized, e.g., ambient air quality has been an issue for years in the town of Kiviõli, but national monitoring cannot reflect the problem as there is no monitoring station [73]. According to our models based on emission data, high concentrations of phenol in Kiviõli can be expected (Figure 1). At a nearby distance (~1 km) from the oil shale chemical and energy complex in Kiviõli and Kohtla-Järve, increased concentrations of phenols, NO_2_, H_2_S, SO_2_, dust, Pb, benzene, formaldehyde etc., have been observed [6]. Phenols, as well as other oil shale chemical products have been shown to be toxic also in earlier studies [74,75]. 

The problem of industrial activities causing local and diffuse contamination, to an extent considerably hazardous to human health, is not unique to Estonia. The effects of industry are large Europe-wide, with the European Environmental Agency reporting industrial emissions as being responsible for damage at a cost of EUR_2005_ 329–1053 billion in Europe between 2008 and 2012, although only a small number of industries are responsible for causing the majority of the costs to people’s health and the environment [45]. Episodes of very high levels of air pollutants can occur that are related to increased mortality and morbidity, especially from specific diseases, as has recently been shown in the industrial city of Ostrava [76]. 

In addition to the direct health effects of industrial air pollution, the annoyance caused by, for example, benzene and the unpleasant odour of hydrogen sulphide needs further attention in the Ida-Viru region. According to the WHO, people can start to smell the (‘rotten egg’) odour from a hydrogen sulphide concentration of 11 µg/m^3^ [77]. As people’s sense of smell is individual, some may get the unpleasant odour sensation even at lower concentrations. Residents living at a distance of 1.0–3.5 km from the oil shale chemical and the energy complex at Kiviõli were four times more likely to complain of unpleasant odours, this was 1.8 times for sleep disorders, and 1.3 times for headaches, than people living further away [6].To eliminate the specific odour, shale oil plants (VKG Group, and Kiviõli Oil Shale Processing and Chemicals Plant) have begun to take measures to ameliorate the situation, especially by improving the hermetic sealing of their various containers. Despite these achievements, the level of annoyance has remained high or even increased. Compared to several earlier studies, the proportion of annoyed people in Ida-Viru County is now much higher [55,56]. This may be explained by the increased expectations of residents regarding the state of the environment [57]. However, also the perception of risk and the related beliefs about pollution and its health effects may provoke annoyance. One important message from here is that care should be taken while informing people about the health effects of the exposure, since health risk perception may induce additional annoyance.

Often health, the environment, and social aspects are strongly interconnected, which make it difficult to separate the effects of environmental pollution from other risk factors. Besides environmental concerns, Ida-Viru County has multiple problems, with rapid population decline, a smaller proportion of young people than average, and the lowest birth rate and life expectancy in Estonia [73,78]. In a study series conducted more than two decades ago by Etlin [6] in the Ida-Viru region, they found a statistically significant (*p* < 0.05) difference between many health indicators among the people living in the area of oil shale chemical and energy enterprises, compared to those living in control areas (outside the oil shale industry). In their study, special attention was paid to the homogeneity of the population groups observed in these areas, to reduce the effect of confounding factors. Therefore, the different quality of the ambient air was regarded as the main reason for the differences in the health indicators between the groups [6]. One of the strengths of the current study has been the possibility to adjust the analysis for gender, age, BMI, education, environmental tobacco smoke, smoking history, and income during the past 12 months to lessen the effect of confounding factors.

The study indicated that working in the oil shale industry poses a significant occupational hazard. Respondents who had been working in the oil shale sector had a significantly higher prevalence of respiratory and cardiovascular diseases. One reason could be high benzene concentrations, which have been found to be twice as high in mines compared to other production units. As the ventilation systems in the mines are weak and raw material is handled with machines using diesel fuel, mine workers had a much higher exposure to benzene than those who worked on the ground [7]. Exposure to diesel engine exhaust gases has also caused alterations of porphyrin and heme metabolism in miners’ peripheral lymphocytes [8]. This may refer to weakening immune mechanisms in people exposed to industrial air pollution components, and such chronic exposure might cause significant disorders in heme synthesis and metabolism [79]. 

As shale oil production has been modernised in several plants in Estonia, the levels of pollutants in Estonia could be lower now than 15–20 years ago. For instance, the B(a)P levels of a modern Finnish coke oven are nearly five times lower compared to older types and also better personal protective equipment is now used [80]. However, some plants in Kiviõli still use older types of coke ovens originating from the 1960s, which release comparable levels of benzo(a)pyrenes as during the earlier studies [6], since the technology is similar. Several health problems are also related to oil shale mining. Recent results show that mine workers still retire relatively early: 58% at an age of 45–49 and 23% at an age of 50–54 [81]. It should be considered that they are entitled to an early pension. Where possible, miners continue to work after retirement age, either doing the same job (if their health allows), or another type of job. Health problems are the main reason for their leaving the job, because according to miners, the damage to their health is often so extensive that they cannot continue working in the mines [81]. Earlier studies have shown mine workers are exposed to high levels of dust [82] and often they had pulmonary diseases caused by inhaling dust [83]. Currently, better self-protective equipment is generally used, but people retiring now or who are presently disabled were still exposed to high concentrations of pollutants during the 1970s–2000s. 

The main limitations of the study include the cross-sectional nature of the investigation, using only current exposure and self-reporting of health-related symptoms and illnesses. In addition, in questionnaire studies indigenous residents, females and older people tend to answer more frequently. The study could be further developed to address the earlier exposures that may have influenced the occurrence of chronic diseases. In addition to exposure assessment in the current study, we would foresee a population-based cohort study of the same exposures, allowing for the analysis of the duration of exposure on the health outcomes. Furthermore, in 10 years the SOHOS study respondents could be included in the follow-up as was the case in the RHINE study [53]. This would also enable us to study the incidence of the respiratory symptoms and chronic diseases. The future studies could also include children to see the effect of the current environmental situation on respiratory health. Methodological developments, e.g., medical screening in combination with interviews could be employed to avoid the possible underreporting of negative effects arising from community pollution by people working in the industry. Whereas the use of control areas was a clear strength of the current study, in future analysis it would be worth investing in gathering data to be able to validate the samples with the health characteristics of the general population. 

## 5. Conclusions

A very large proportion of residents of Ida-Viru County have been exposed to oil shale industries and more than one quarter have worked in the sector. Residents of Ida-Viru County reported higher rates of four respiratory symptoms (chest tightness, wheezing, long-term cough, shortness of breath) and five serious chronic health states including hypertension and heart disease than Estonian reference populations. Several of these self-reported health data were statistically significantly associated with PM_2.5_, benzene and phenol exposure. Perceived health risk from air pollution significantly contributes to annoyance from air pollution. Annoyed people have higher odds of experiencing respiratory symptoms and lower self-assessed health.

The results of this study show that we need to pay more attention to the state of the environment and the health of residents and industry workers in the region, and to carry out even more comprehensive health surveys, also including children. The long-term improvement of the health of people living in Ida-Viru County depends on cooperation between decision-makers, scientists, local government, businesses, health systems and local residents.

## Figures and Tables

**Table 1:** Socio-demographic characteristics of the respondents, *n* (%).

	Ida-Viru County	Lääne-Viru County	Tartu	Total
**Gender**				
Female	508 (59.8)	105 (57.7)	1430 (60)	2046 (59.9)
**Age**				
18–27	78 (9.2)	20 (11)	277 (11.6)	376 (11)
28–37	105 (12.4)	28 (15.5)	462 (19.4)	598 (17.5)
38–47	171 (20.2)	34 (18.7)	949 (39.8)	1151 (33.7)
48–57	204 (24)	48 (26.6)	467 (19.6)	717 (21)
58–67	241 (28.4)	38 (21)	224 (9.4)	505 (14.8)
68+	49 (5.8)	13 (7.2)	5 (0.2)	68 (2)
**Body mass index (BMI)**				
Underweight (BMI < 20)	62 (7.3)	10 (5.6)	231 (9.7)	304 (8.9)
Healthy (BMI 20–24.99	254 (29.9)	61 (33.7)	1020 (42.8)	1339 (39.2)
Overweight (BMI 25–30)	306 (36)	64 (34.9)	713 (29.9)	1079 (31.6)
Obese (BMI > 30)	228 (26.8)	47 (25.8)	420 (17.6)	693 (20.3)
**Ethnicity**				
Estonian	272 (32)	153 (84)	2026 (85)	2179 (63.8)
Russian	517 (60.9)	21 (11.6)	312 (13.1)	1093 (32)
Other	60 (7.1)	8 (4.4)	45 (1.9)	143 (4.2)
**Monthly net income during the last 12 months per family member**				
Under 100 €	39 (4.6)	2 (1.2)	43 (1.8)	99 (2.9)
100–299 €	246 (29)	33 (18.1)	322 (13.5)	686 (20.1)
300–499 €	301 (35.4)	67 (36.7)	665 (27.9)	1079 (31.6)
500–799 €	161 (19)	52 (28.8)	701 (29.4)	861 (25.2)
800–999 €	55 (6.5)	8 (4.5)	336 (14.1)	348 (10.2)
Over 1000 €	47 (5.5)	19 (10.7)	317 (13.3)	342 (10)
**Education**				
Elementary or basic	79 (9.3)	21 (11.6)	122 (5.1)	222 (6.5)
Secondary	540 (63.6)	121 (66.3)	1082 (45.4)	1745 (51.1)
Applied or higher	230 (27.1)	40 (22.1)	1180 (49.5)	1448 (42.4)
**Working in oil shale sector**				
Have been working	239 (28.2)	1 (0.5)	48 (2)	287 (8.4)
**Smoking**				
Current smoker	212 (25)	39 (21.3)	460 (19.3)	710 (20.8)
Ex-smoker	179 (21.1)	37 (20.2)	434 (18.2)	649 (19)
**Self-assessed health status**				
Very good	30 (3.5)	11 (6.1)	384 (16.1)	345 (10.1)
Good	241 (28.4)	71 (39.2)	1321 (55.4)	1468 (43)
Average	456 (53.7)	78 (43.1)	594 (24.9)	1305 (38.2)
Bad	112 (13.2)	20 (11)	79 (3.3)	273 (8)
Very bad	11 (1.3)	1 (0.6)	10 (0.4)	27 (0.8)
**Marital status**				
Single	113 (13.3)	38 (20.8)	432 (18.1)	584 (17.1)
Married	464 (54.7)	84 (46.1)	1087 (45.6)	1636 (47.9)
Cohabiting	122 (14.4)	37 (20.2)	620 (26)	779 (22.8)
Divorced	94 (11.1)	16 (9)	210 (8.8)	321 (9.4)
Widowed	55 (6.5)	7 (4)	33 (1.4)	99 (2.9)

**Table 2:** The prevalence of respiratory and cardiovascular diseases in different counties and among ethnic groups, *n* (%).

	Ida-Viru County	Lääne-Viru County	Tartu	Estonians	Non-Estonians
**During the last 12 months**					
Wheezing without a cold	**358 (42.2 ^1^)**	66 (36.2)	427 (17.9)	405 (30.0)	**353 (46.0 ^3^)**
Chest tightness	**256 (30.1 ^1^)**	42 (23.3)	403 (16.9)	215 (15.9)	**242 (31.6 ^3^)**
Shortness of breath	**165 (19.4 ^1^)**	27 (14.6)	286 (12.0)	143 (10.6)	**156 (20.4 ^3^)**
Attack of coughing	**384 (45.2)**	77 (42.2)	997 (41.8)	539 (39.9)	324 (42.3)
Asthma attack	**65 (7.6 ^1^)**	12 (6.7)	91 (3.8)	62 (4.6)	**60 (7.8 ^3^)**
**Ever had**					
Wheezing	**275 (32.4 ^1,2^)**	37 (20.5)	539 (22.6)	251 (18.6)	**254 (33.1 ^3^)**
Long-term cough	**249 (29.3 ^1^)**	41 (22.3)	498 (20.9)	259 (19.2)	**242 (31.6 ^3^)**
Nose allergies/hay fever	185 (21.8)	41 (22.5)	577 (24.2)	320 (23.7)	157 (20.5)
Asthma	64 (7.5)	15 (8.0)	174 (7.3)	91 (6.7)	58 (7.5)
COPD	20 (2.4)	3 (1.7)	24 (1.0)	12 (0.9)	**21 (2.7 ^3^)**
Hypertension	**292 (34.4 ^1^)**	59 (32.2)	370 (15.5)	181 (13.4)	**250 (32.6 ^3^)**
Heart disease	**138 (16.3 ^1,2^)**	18 (10.0)	241 (10.1)	85 (6.3)	**130 (16.9 ^3^)**
Myocardial infarction	**66 (7.8 ^1^)**	7 (4.1)	48 (2.0)	16 (1.2)	**61 (7.9 ^3^)**
Stroke	**31 (3.6 ^1^)**	4 (2.4)	24 (1.0)	11 (0.8)	**31 (4.1 ^3^)**
Diabetes	**84 (9.9 ^1^)**	11 (5.8)	69 (2.9)	35 (2.6)	**72 (9.4 ^3^)**

^1^ Respondents from Ida-Viru County different (*p* < 0.05) from respondents from Tartu; ^2^ Respondents from Ida-Viru County different (*p* < 0.05) from respondents from Lääne-Viru County; ^3^ Non-Estonian respondents different (*p* < 0.05) from Estonian respondents; Values in bold: *p* < 0.05.

**Table 3:** Odds ratios (95% CI) on disease prevalence per interquartile change in pollutants concentrations.

	PM_2.5_	Benzene	Phenol
During the last 12 months *			
Wheezing without a cold	1.03 (0.92–1.14)	1.06 (0.79–1.43)	1.36 (0.98–1.90)
Chest tightness	**1.13 (1.02–1.26)**	1.30 (0.96–1.76)	**1.44 (1.03–2.00)**
Shortness of breath	**1.16 (1.03–1.31)**	1.14 (0.80–1.62)	1.18 (0.80–1.74)
Attack of coughing	1.04 (0.94–1.15)	0.94 (0.71–1.24)	0.95 (0.70–1.30)
Asthma attack	**1.22 (1.04–1.42)**	1.14 (0.67–1.95)	0.60 (0.32–1.15)
Ever had *			
Wheezing	1.10 (0.99–1.22)	1.16 (0.86–1.56)	1.11 (0.80–1.55)
Long-term cough	0.99 (0.89–1.11)	1.31 (0.96–1.77)	**1.48 (1.06–2.07)**
Allergic rhinitis	1.07 (0.94–1.22)	0.92 (0.64–1.32)	0.89 (0.59–1.34)
Asthma	1.04 (0.87–1.24)	1.20 (0.70–2.06)	0.84 (0.45–1.56)
COPD	1.22 (0.84–1.76)	1.97 (0.72–2.10)	1.92 (0.71–5.19)
Hypertension	1.10 (0.98–1.24)	1.15 (0.83–1.59)	1.21 (0.84–1.74)
Heart disease	1.12 (0.98–1.28)	1.24 (0.83–1.84)	1.30 (0.85–1.98)
Myocardial infarction	0.99 (0.80–1.22)	**1.98 (1.11–3.53)**	**2.17 (1.23–3.83)**
Stroke	1.01 (0.76–1.35)	1.39 (0.62–3.11)	1.14 (0.50–2.62)
Diabetes	1.01 (0.85–1.21)	1.42 (0.86–2.34)	1.33 (0.78–2.27)

* Logistic regression analysis was adjusted for gender, age, body mass index (BMI), education, environmental tobacco smoke, smoking history, and income during past 12 months; COPD: Chronic obstructive pulmonary disease; Values in bold: *p* < 0.05.

**Table 4:** Association between specific factors, air pollutants and the prevalence of annoyance in the three investigated counties, odds ratios (95% CI).

**Base model**	**Ida-Viru County**	**Lääne-Viru County**	**Tartu**
Gender *(ref females)*	**0.64 (0.42–0.97)**	1.01 (0.36–2.88)	**0.64 (0.41–1.00)**
Age	1.00 (0.99–1.02)	0.99 (0.95–1.03)	0.99 (0.97–1.02)
Elementary or basic education *(ref applied or higher)*	1.13 (0.56–2.27)	0.36 (0.03–4.58)	1.41 (0.59–3.33)
Secondary education *(ref applied or higher)*	0.87 (0.56–1.34)	0.66 (0.21–2.04)	1.21 (0.78–1.87)
Income	1.08 (0.91–1.27)	1.17 (0.74–1.85)	0.89 (0.75–1.05)
Current or ex-smokers *(ref never smokers)*	0.89 (0.70–1.13)	0.50 (0.24–1.05)	1.04 (0.82–1.35)
Self-assessed health status	**1.59 (1.21–2.08)**	**2.56 (1.23–5.36)**	**1.49 (1.13–1.97)**
Pollutants *^,#^			
PM_2.5_	1.07 (0.94–1.22)		
Benzene	1.04 (0.87–1.24)		
Phenol	1.22 (0.84–1.76)		
Social and psychological factors *			
Working in oil shale sector	1.36 (0.86–2.15)	0	**6.13 (2.31–16.28)**
Ethnicity *(ref Estonian)*	1.19 (0.85–1.67)	0.87 (0.31–2.44)	**1.56 (1.03–2.36)**
Perceived risk	**0.35 (0.28–0.44)**	**0.34 (0.2–0.58)**	**0.39 (0.32–0.49)**
Symptoms during the last 12 months *			
Wheezing without a cold	**2.30 (1.31–4.04)**	0.11 (0.01–0.92)	0
Chest tightness	**2.88 (1.91–4.33)**	**3.19 (1.02–9.92)**	**2.26 (1.36–3.74)**
Shortness of breath	1.60 (0.92–2.79)	94.83 (3.35–2680.68)	**3.24 (1.88–5.57)**
Attack of coughing	**1.99 (1.34–2.95)**	2.10 (0.75–5.84)	**1.57 (1.03–2.38)**
Asthma attack	1.48 (0.79–2.77)	4.83 (0.79–29.35)	2.16 (0.98–4.75)
Ever had chronic diseases *			
Wheezing	**1.98 (1.31–2.99)**	1.52 (0.48–4.82)	**1.81 (1.13–2.90)**
Long-term cough	1.29 (0.86–1.94)	3.04 (1.01–9.16) *	**1.92 (1.21–3.05)**
Allergic rhinitis	1.40 (0.82–2.37)	1.36 (0.34–5.45)	1.34 (0.78–2.27)
Asthma	1.18 (0.60–2.32)	2.85 (0.6–13.61)	1.36 (0.65–2.85)
COPD	1.69 (0.56–5.14)	6.21 (0.16–246.71)	3.18 (0.78–12.8)
Hypertension	1.05 (0.68–1.63)	1.13 (0.35–3.69)	1.12 (0.51–2.47)
Heart disease	1.22 (0.61–2.42)	7.81 (1.41–43.16)	0.47 (0.16–1.39)
Myocardial infarction	1.22 (0.61–2.42)	17.32 (1.47–203.29)	0
Stroke	0.69 (0.21–2.24)	0	0
Diabetes	0.67 (0.35–1.29)	0.47 (0.04–4.84)	0.83 (0.09–6.96)

* Logistic regression analysis was adjusted for gender, age, BMI, education, environmental tobacco smoke, smoking history, and income during past 12 months; ^#^ annoyance prevalence per interquartile change in pollutants concentrations; Values in bold: *p* < 0.05.

**Figure A1:**
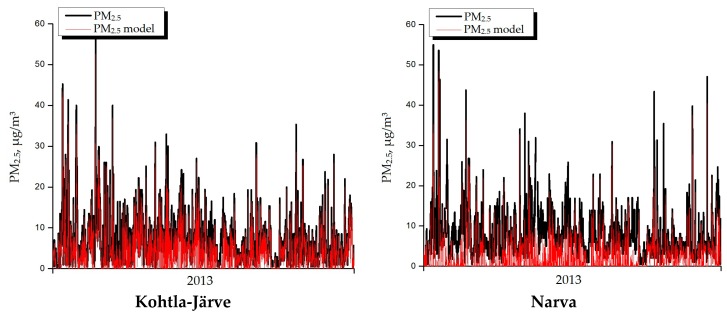

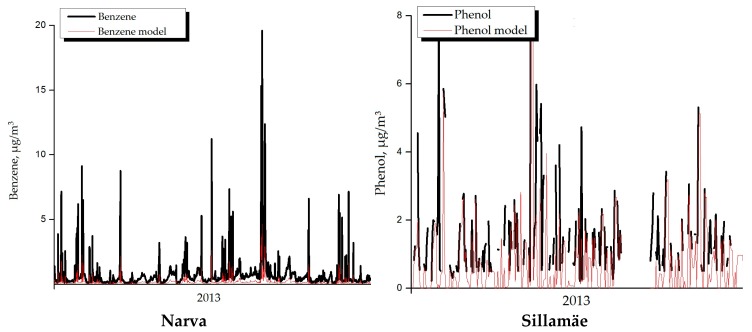
Measured and modelled concentrations of PM_2.5_, benzene and phenol in monitoring stations in 2013.

## Appendix A. Used Questions

From the SOHOS questionnaire we used the following questions:

**Gender**—“Are you male or female?”
**Age**—Calculation based on “What is your date of birth?”, and “What is today’s date?”
**Body mass index (BMI)**—calculated as (weight (kg))/height^2^ (m)) based on “How much do you weigh?”, and “How tall are you?”
**Ethnicity**—“What ethnicity you belong to?”
**Education**—“Please mark the educational level which best describes your level.”
**Self-assessed health status**—“How would you rate your health today?”
**Marital status**—“What is your current marital status?”
**Current smoker**—“Do you smoke? (this applies even if you only smoke the odd cigarette/cigar or pipe every week)”
**Ex-smoker**—“Did you smoke previously?”
**Monthly net income during the last 12 months per family member**—“How large was the monthly net income during the last 12 months per family member”
**Wheezing without a cold**—“Have you had wheezing or whistling in your chest at any time in the last 12 months?”, and if so, “Have you had this wheezing or whistling when you did not have a cold?”
**Chest tightness**—“Have you woken up with a feeling of tightness in your chest at any time in the last 12 months?”
**Shortness of breath**—“Have you been woken by an attack of shortness of breath at any time in the last 12 months?”
**Attack of coughing**—“Have you been woken by an attack of coughing at any time in the last 12 months?”
**Asthma attack**—“Have you had an attack of asthma in the last 12 months?”
**Wheezing**—“Have you ever had wheezing or whistling in your chest?”
**Long-term cough**—“In recent years, have you been troubled by a protracted cough?”
**Allergic rhinitis**—“Have you ever experienced nasal symptoms such as nasal congestion, rhinorrhoea (runny nose) and/or sneezing attacks without having a cold?”, and if “*Yes*”; “*Yes*” to question “Do you have any nasal allergies including hay fever?”
**Asthma**—“Have you ever had asthma diagnosed by a doctor?”
**COPD**—“Has a doctor ever told you that you have chronic obstructive pulmonary disease (COPD)?”
**Hypertension**—“Have you ever had hypertension (high blood pressure) diagnosed by a doctor?”
**Heart diseases**—“Do you have any heart disease?”
**Myocardial infarction**—“Have you ever been treated in hospital because of heart infarction?”
**Stroke**—“Have you ever had stroke?”
**Diabetes**—“Have you ever had diabetes diagnosed by a doctor?”
**Smoking history**—“Are you a smoker?” and “Are you an ex-smoker?”; defining “never smokers”, “current smokers” and “ex-smokers”.
**Annoyance with air pollution**—“How annoyed are you due to air pollution (from traffic, industry etc.) if you have opened the window?

Not at all annoying 1 2 3 4 5 6 7 8 9 10 Intolerably annoying.

## Appendix C.

**Table A1:** Odds ratios (95% CI) on disease prevalence per interquartile change in pollutants concentrations.

**Crude Model**			
	**PM_2.5_**	**Benzene**	**Phenol**
**During the last 12 months**			
Wheezing without a cold	1.03 (0.94–1.13)	1.06 (0.98–1.38)	1.28 (0.95–1.71)
Chest tightness	**1.11 (1.01–1.22)**	1.28 (0.97–1.67)	**1.41 (1.05–1.88)**
Shortness of breath	1.09 (0.98–1.22)	1.26 (0.92–1.73)	1.21 (0.86–1.70)
Attack of coughing	1.03 (0.94–1.13)	0.90 (0.84–1.15)	0.89 (0.68–1.17)
Asthma attack	1.20 (1.04–1.38)	1.24 (0.93–2.00)	0.64 (0.36–1.12)
**Ever had**			
Wheezing	1.07 (0.98–1.18)	**1.31 (1.00–1.71)**	1.22 (0.91–1.64)
Long-term cough	1.00 (0.91–1.11)	**1.32 (1.00–1.74)**	**1.42 (1.06–1.91)**
Allergic rhinitis	1.03 (0.92–1.16)	0.89 (0.64–1.23)	0.86 (0.59–1.26)
Asthma	1.11 (0.96–1.30)	1.39 (0.85–2.26)	0.80 (0.46–1.39)
COPD	1.09 (0.84–1.41)	1.59 (0.69–1.77)	2.07 (0.90–4.76)
Hypertension	1.03 (0.94–1.13)	1.16 (0.89–1.51)	1.21 (0.90–1.62)
Heart disease	1.07 (0.95–1.20)	**1.44 (1.01–2.05)**	**1.53 (1.06–2.21)**
Myocardial infarction	0.95 (0.79–1.14)	**2.03 (1.22–3.39)**	**2.23 (1.36–3.64)**
Stroke	0.95 (0.73–1.24)	1.32 (0.65–2.67)	1.34 (0.65–2.78)
Diabetes	0.97 (0.83–1.13)	1.42 (0.92–2.19)	1.33 (0.85–2.10)
**Only Estonians in Ida-Viru County**			
	**PM_2.5_**	**Benzene**	**Phenol**
**During the last 12 months ***			
Wheezing without a cold	0.96 (0.76–1.21)	0.96 (0.56–1.64)	0.97 (0.83–1.21)
Chest tightness	1.17 (0.93–1.46)	1.27 (0.72–2.23)	1.07 (0.92–1.23)
Shortness of breath	1.23 (0.95–1.59)	**2.36 (1.20–2.04)**	1.12 (0.95–1.31)
Attack of coughing	1.08 (0.88–1.33)	0.96 (0.59–1.56)	0.96 (0.84–1.09)
Asthma attack	1.25 (0.89–1.77)	1.12 (0.68–1.68)	0.99 (0.71–1.37)
**Ever had ***			
Wheezing	1.05 (0.84–1.32)	1.17 (0.67–2.04)	0.96 (0.83–1.12)
Long-term cough	1.24 (0.98–1.58)	1.09 (0.61–1.96)	1.01 (0.86–1.18)
Allergic rhinitis	1.00 (0.76–1.32)	0.63 (0.62–1.19)	0.76 (0.59–0.97)
Asthma	0.78 (0.48–1.27)	0.93 (0.66–2.52)	1.03 (0.81–1.31)
COPD	-	-	0.40 (0.05–3.36)
Hypertension	1.14 (0.89–1.47)	1.49 (0.79–2.83)	**1.19 (1.00–1.40)**
Heart disease	0.96 (0.65–1.42)	0.93 (0.67–2.25)	1.06 (0.85–1.31)
Myocardial infarction	0.96 (0.48–1.92)	1.38 (0.67–2.19)	1.23 (0.88–1.72)
Stroke	0.83 (0.09–7.58)	0.55 (0.13–2.06)	0.91 (0.48–1.72)
Diabetes	0.83 (0.49–1.40)	1.21 (0.70–1.90)	0.95 (0.67–1.34)
**Only non-Estonians in Ida-Viru County**			
	**PM_2.5_**	**Benzene**	**Phenol**
**During the last 12 months ***			
Wheezing without a cold	1.04 (0.91–1.18)	1.03 (0.77–1.38)	**1.52 (1.02–2.25)**
Chest tightness	1.09 (0.96–1.25)	1.11 (0.82–1.49)	1.35 (0.91–2.01)
Shortness of breath	1.13 (0.98–1.31)	0.81 (0.58–1.13)	0.96 (0.61–1.52)
Attack of coughing	1.05 (0.93–1.19)	1.02 (0.77–1.36)	1.05 (0.72–1.53)
Asthma attack	**1.22 (1.02–1.46)**	0.99 (0.61–1.61)	**0.47 (0.23–0.98)**
**Ever had ***			
Wheezing	1.11 (0.97–1.26)	1.02 (0.77–1.36)	1.08 (0.73–1.59)
Long-term cough	0.92 (0.80–1.05)	1.14 (0.85–1.53)	1.41 (0.96–2.08)
Allergic rhinitis	1.12 (0.95–1.31)	1.15 (0.80–1.66)	1.15 (0.71–1.88)
Asthma	1.10 (0.90–1.35)	1.11 (0.65–1.86)	0.66 (0.32–1.37)
COPD	1.15 (0.77–1.72)	1.26 (0.54–2.92)	1.82 (0.59–5.59)
Hypertension	1.09 (0.95–1.26)	0.93 (0.68–1.28)	0.92 (0.60–1.42)
Heart disease	1.15 (0.98–1.35)	1.01 (0.70–1.47)	1.02 (0.62–1.67)
Myocardial infarction	0.96 (0.76–1.22)	1.49 (0.88–2.50)	1.74 (0.92–3.29)
Stroke	0.99 (0.72–1.35)	1.11 (0.57–2.16)	0.85 (0.35–2.09)
Diabetes	1.04 (0.84–1.27)	1.26 (0.79–2.03)	1.22 (0.66–2.24)
**Only those, who have been working in oil shale sector**			
	**PM_2.5_**	**Benzene**	**Phenol**
**During the last 12 months ***			
Wheezing without a cold	**1.33 (1.05–1.70)**	1.03 (0.63–1.69)	1.08 (0.75–1.57)
Chest tightness	1.10 (0.87–1.40)	1.47 (0.88–2.44)	1.37 (0.95–1.97)
Shortness of breath	1.24 (0.96–1.61)	0.89 (0.49–1.59)	1.15 (0.75–1.76)
Attack of coughing	1.02 (0.82–1.28)	1.08 (0.67–1.72)	0.98 (0.69–1.38)
Asthma attack	**1.93 (1.30–2.86)**	2.52 (0.83–7.63)	0.61 (0.26–1.45)
**Ever had ***			
Wheezing	**1.36 (1.06–1.74)**	1.06 (0.65–1.73)	0.87 (0.60–1.27)
Long-term cough	0.99 (0.77–1.28)	1.30 (0.77–2.19)	1.41 (0.97–2.07)
Allergic rhinitis	1.27 (0.88–1.82)	0.99 (0.49–2.02)	0.86 (0.49–1.51)
Asthma	**1.47 (1.01–2.15)**	**3.50 (1.01–12.11)**	0.84 (0.55–1.27)
COPD	0.55 (0.04–7.84)	2.16 (0.26–17.91)	1.33 (0.37–4.70)
Hypertension	1.27 (0.97–1.67)	1.00 (0.58–1.72)	0.89 (0.59–1.32)
Heart disease	1.11 (0.82–1.50)	1.26 (0.68–2.36)	1.30 (0.84–2.02)
Myocardial infarction	0.71 (0.38–1.32)	2.26 (0.95–5.37)	1.60 (0.91–2.81)
Stroke	0.51 (0.20–1.27)	0.74 (0.26–2.12)	1.39 (0.72–2.67)
Diabetes	1.20 (0.86–1.68)	1.58 (0.72–3.48)	0.95 (0.65–1.38)
**Only those, who have not been working in oil shale sector**			
	**PM_2.5_**	**Benzene**	**Phenol**
**During the last 12 months ***			
Wheezing without a cold	0.97 (0.84–1.12)	0.99 (0.68–1.44)	1.24 (0.82–1.87)
Chest tightness	**1.17 (1.02–1.35) ***	1.30 (0.88–1.91)	1.30 (0.86–1.96)
Shortness of breath	1.15 (0.98–1.35)	1.26 (0.80–1.99)	1.11 (0.68–1.81)
Attack of coughing	1.03 (0.90–1.18)	0.91 (0.64–1.28)	0.91 (0.62–1.34)
Asthma attack	1.07 (0.86–1.34)	0.76 (0.41–1.41)	0.58 (0.27–1.25)
**Ever had ***			
Wheezing	1.04 (0.90–1.19)	1.13 (0.78–1.64)	1.14 (0.76–1.72)
Long-term cough	0.98 (0.85–1.13)	1.34 (0.92–1.95)	1.33 (0.89–2.00)
Allergic rhinitis	1.05 (0.90–1.24)	0.80 (0.52–1.24)	0.74 (0.45–1.21)
Asthma	0.91 (0.69–1.18)	0.81 (0.42–1.55)	0.72 (0.33–1.57)
COPD	1.30 (0.88–1.92)	1.98 (0.62–6.31)	1.87 (0.62–5.64)
Hypertension	1.04 (0.89–1.21)	1.16 (0.77–1.74)	1.34 (0.86–2.10)
Heart disease	1.25 (0.92–1.35)	1.24 (0.73–2.11)	1.23 (0.70–2.16)
Myocardial infarction	1.08 (0.82–1.43)	2.07 (0.93–1.06)	**2.26 (1.04–4.88) ***
Stroke	1.08 (0.69–1.69)	3.85 (0.85–17.49)	1.85 (0.54–6.28)
Diabetes	0.88 (0.65–1.19)	1.25 (0.66–2.40)	0.91 (0.45–1.84)

* Logistic regression analysis was adjusted for gender, age, BMI, education, environmental tobacco smoke, smoking history, and income during past 12 months; Values in bold: *p* < 0.05.

## Appendix D.

**Table A2:** High annoyance (%) due to air pollution * per demographics and health problems of people in Ida-Viru County and the control regions (Lääne-Viru and Tartu), and regional comparisons.

	Ida-Viru County	Lääne-Viru County	Tartu	Chi^2^	*p* Value
**Total**	22.4	14.9	11.8	36.70	**0.000**
**Gender**					
Male	22.4	14.9	11.8	36.70	**0.000**
Female	25.5	15.6	13.2	28.25	**0.006**
**Age**					
18–27	19.5	20.0	13.3	2.24	0.327
28–37	24.5	11.1	11.0	12.68	**0.002**
38–47	18.4	9.1	10.1	7.15	**0.028**
48–57	24.5	15.6	46.2	5.28	0.071
58–67	24.3	20.0	22.2	0.31	0.855
68+	17.5	14.3	0	0.65	0.721
**Ethnicity**					
Estonian	18.5	13.7	10.0	13.70	**0.001**
Non-Estonian	24.2	17.9	21.9	0.85	0.655
**Monthly average net income during the last 12 months per family member**					
Under 100 €	26.5	50.0	21.1	0.85	0.655
100–299 €	22.3	10.3	15.4	4.08	0.130
300–499 €	22.8	15.3	15.0	5.93	0.052
500–799 €	15.2	12.5	9.0	3.95	0.139
800–999 €	28.8	25.0	9.5	11.92	**0.003**
Over 1000 €	28.6	16.7	8.2	11.62	**0.003**
**Education**					
Elementary or basic	25.4	13.3	16.7	2.00	0.369
Secondary	20.9	14.2	13.1	10.70	**0.005**
Applied or higher	24.2	17.9	10.3	24.59	**0.000**
**Self-assessed health status**					
Very good	14.3	23.1	6.1	6.17	**0.046**
Good	17.3	7.2	10.1	9.70	**0.008**
Average	21.5	16.2	17.2	1.74	0.420
Bad	37.9	10.9	27.0	1.37	0.504
Very bad	20.0	0	0	35.50	0.000
**During the last 12 months**					
Chest tightness	36.6	27.0	22.9	8.46	**0.015**
Shortage of breath	38.4	34.8	28.8	2.49	0.288
Asthma attack	33.3	40.0	21.4	2.27	0.321
Ever had					
Wheezing	34.0	22.9	21.7	7.80	**0.020**
Long-term cough	28.9	25.0	19.8	4.82	0.090
Allergic rhinitis	25.8	18.8)	15.3	6.54	**0.038**
Asthma	31.7	36.4	12.3	8.02	**0.018**
COPD	30.0	33.3	30.0	0.01	0.993

* Respondents who rated 7–10 on a 10-point scale, ranging from no annoyance (1) to very high annoyance (10).
